# Oral pre‐exposure prophylaxis initiation, continuation and adherence among pregnant and postpartum women receiving antenatal and postnatal care: a systematic review

**DOI:** 10.1002/jia2.70035

**Published:** 2025-10-08

**Authors:** Anke Rotsaert, Zaynab Essack, Shannon Bosman, Dvora Joseph Davey, Bernadette Hensen

**Affiliations:** ^1^ Department of Public Health Institute of Tropical Medicine Antwerp Belgium; ^2^ Centre for Community‐Based Research Human Sciences Research Council Pietermaritzburg South Africa; ^3^ South African Research Ethics Training Initiative University of KwaZulu‐Natal Natal Durban South Africa; ^4^ Department of Infectious Diseases Geffen School of Medicine University of California Los Angeles California USA; ^5^ Department of Epidemiology & Biostatistics School of Public Health University of Cape Town Cape Town South Africa

**Keywords:** antenatal care, postpartum period, pre‐exposure prophylaxis, pregnancy, review, women

## Abstract

**Introduction:**

In 2023, one‐fourth of new HIV acquisitions in children globally resulted from vertical transmission following incident HIV during pregnancy or breastfeeding. Oral pre‐exposure prophylaxis (PrEP) with tenofovir disoproxil and emtricitabine is safe and effective in pregnancy and postpartum, with long‐acting options emerging. Integrating PrEP into antenatal and postnatal care (ANC/PNC) is a crucial person‐centred approach to prevent maternal HIV acquisition and vertical transmission. This review summarizes oral PrEP initiation, continuation and adherence among pregnant and postpartum women receiving ANC/PNC.

**Methods:**

We systematically searched three databases for English‐language quantitative studies published between 1 January 2015 and 28 March 2024. Eligible studies focused on pregnant and/or postpartum women accessing PrEP through ANC/PNC, and reported on initiation (receipt of prescription or self‐reported use), continuation (persistent use over time) and/or adherence (self‐reported and/or objective).

**Results:**

We identified 481 articles; 12 studies from Kenya, Lesotho, Malawi and South Africa met our inclusion criteria. Study heterogeneity (e.g. definitions used, population included, follow‐up time) precluded meta‐analysis. All studies enrolled pregnant women; three also enrolled postpartum women. Median gestational age at enrolment ranged from 20 to 26 weeks, and follow‐up periods from 1 month post‐enrolment to 12 months postpartum. Oral PrEP initiation ranged from 14% to 84%. Continuation at 3 months ranged from 22% to 90% and declined postpartum in all studies. Self‐reported adherence (daily use) ranged from 11% to 81% in the past 7 or 30 days at 1 month (four studies) and from 54% to 81% at 3 months (two studies). Objectively measured adherence ranged from 34% to 62% for detectable tenofovir or tenofovir diphosphate levels at 1 month (three studies). One Kenyan trial demonstrated that universal versus risk‐based offers of oral PrEP resulted in similar PrEP use and HIV incidence. Two‐way SMS communication (Kenya) and real‐time adherence biofeedback counselling using urine tenofovir testing (South Africa) enhanced PrEP continuation/adherence compared to standard‐of‐care.

**Discussion:**

Integrating oral PrEP into ANC/PNC showed high initiation among pregnant/postpartum women; however, continuation and adherence were suboptimal.

**Conclusions:**

Oral PrEP integration into ANC/PNC can reach pregnant/postpartum women. Maximizing its impact will require offering long‐acting PrEP, person‐centred interventions to support adherence/continued use and differentiated delivery responsive to women's needs.

**PROSPERO Number:**

CRD42024513442

## 1 INTRODUCTION

In 2023, women and girls constituted 44% of all individuals who newly acquired HIV worldwide [1]. Women's risk of acquiring HIV is elevated during pregnancy and postpartum [2, 3]. This elevated risk is attributable to a combination of biological, behavioural and social factors, including changes in the vaginal microbiome [4]. Acquiring HIV during pregnancy or postpartum not only poses a threat to maternal health, but also increases the risk of vertical HIV transmission [4]. In 2023, one in four new HIV acquisitions in children globally resulted from mothers acquiring HIV during pregnancy or breastfeeding [5]. HIV prevention among pregnant and postpartum women is a global health priority and a key pillar to ending HIV‐related deaths among children by 2030 [6].

Oral pre‐exposure prophylaxis (PrEP) with tenofovir disoproxil and emtricitabine is an efficacious biomedical HIV prevention tool. In settings with high HIV burden, the World Health Organization (WHO) recommends PrEP for pregnant and postpartum women who are HIV negative [4]. Although PrEP rollout is expanding globally, with 7.5 million individuals having initiated PrEP by the second quarter of 2024, initiation among pregnant or postpartum women remains limited [7, 8, 9]. Evidence from a systematic review confirmed that oral PrEP is safe for use during pregnancy and postpartum, making it crucial to expand use among pregnant and postpartum women [10].

The integration of provider‐initiated HIV testing and counselling into antenatal care (ANC) increased the diagnosis of pregnant women living with HIV and improved access to comprehensive care [11]. This serves as a valuable model for the integration of PrEP services in antenatal and postnatal care (ANC/PNC), an approach recommended by WHO [4, 12]. Given that 88% of pregnant adolescent girls and women aged 15–49 years worldwide attended at least one ANC visit in 2023 [13], and 71% of adolescent girls and women received routine PNC within 2 days after birth [14], integrating and delivering oral PrEP as part of routine ANC/PNC is a promising approach to reach pregnant and postpartum women [11, 13]. Evidence on how to operationalize integration remains sparse and mainly stems from a few African countries, primarily Kenya and South Africa [15]. This available evidence suggests that daily PrEP use by pregnant and postpartum women presents unique challenges (e.g. concerns of impact on infant development), with substantial early discontinuation being reported [16]. Factors influencing PrEP use include side effects, perceived risk of HIV acquisition, limited partner and family support, or negative endorsement, or limited knowledge among providers [16, 17, 18], with structural barriers related to logistics, transport and affordability to take PrEP [15, 19, 20, 21].

To enhance understanding of oral PrEP use among pregnant and postpartum women globally, this systematic review aims to provide a comprehensive overview of oral PrEP initiation, continuation and adherence among pregnant and postpartum women receiving ANC/PNC. These findings aim to inform strategies for delivering oral PrEP through person‐centred and differentiated service delivery approaches. These approaches prioritize the unique needs and preferences of women during pregnancy and postpartum and facilitate access by integrating PrEP services with essential maternal healthcare services that women already attend. These insights on oral PrEP use will inform how emerging long‐acting PrEP options, such as the dapivirine ring [22], injectable lenacapavir [23] and cabotegravir [24, 25]—supported by evidence of safety and efficacy in pregnant and postpartum women—may complement oral PrEP by offering greater choice and longer efficacy of product compared to daily use.

## 2 METHODS

This systematic review addressed the research question: “What are the initiation rates of oral PrEP, the continuity of its use, and adherence levels among pregnant and postpartum women when administered through antenatal and postnatal care?”, and was conducted in accordance with the Preferred Reporting Items for Systematic Review and Meta‐Analyses (PRISMA) guidelines [26]. The study was registered on PROSPERO (ID: 2024 CRD42024513442) [27].

### 2.1 Search strategy and selection criteria

We searched PubMed, Web of Science Core Collection and Global Health covering the period 1 January 2015 (following WHO recommendations on oral PrEP) to 28 March 2024. Database searches were performed on 28 March 2024. We used the following core search terms across all databases: (hiv OR “human immunodeficiency virus”) AND (“postpartum period” OR postpartum OR pregnan* OR perinatal OR breastfeed* OR lactati*) AND (wom?n OR “adolescent girl” OR “adolescent girls” OR “young women” OR “young woman” OR female) AND (“pre‐exposure prophylaxis” OR “preexposure prophylaxis” OR “prep”). Appendix S1 provides details of the search strategy for each database. While we had no geographical restrictions, we restricted our selection to English articles. Studies were eligible if they were published in peer‐reviewed journals, used a quantitative study design (e.g. observational and (quasi)experimental studies), focused on pregnant and/or postpartum women accessing PrEP through ANC/PNC and reported on one or more of the specified outcomes: initiation of PrEP (e.g. prescription receipt at the first or any ANC/PNC visit or self‐reported use at a follow‐up visit after accepting PrEP at a prior ANC/PNC visit), continuation of PrEP (persistent use at each visit, e.g. self‐reporting continued use, prescription receipt or attendance follow‐up visit where PrEP was dispensed) and adherence to PrEP (e.g. self‐reported or objectively measured through blood or urine samples in the previous 7 or 30 days). Studies exploring willingness to use PrEP or assessing use outside of clinics offering ANC/PNC, including maternal and child health clinics, were excluded. Additionally, we conducted a secondary search for references in all eligible studies. Conference abstracts were not considered as they often lack sufficient methodological detail around outcome definitions and study context that were needed for our assessments of risk of bias and data synthesis.

### 2.2 Study selection

**Figure 1:**
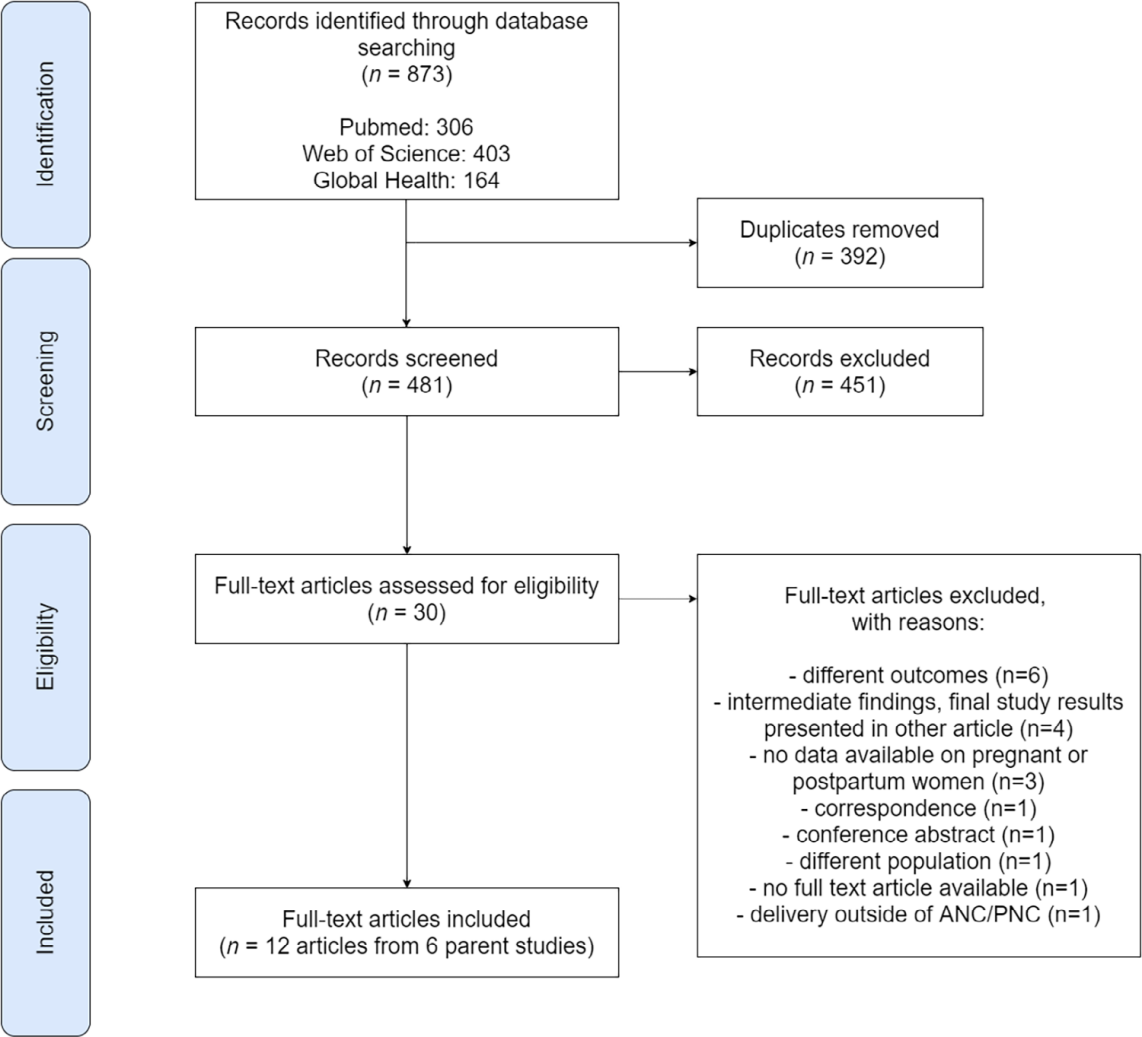
Prisma flowchart for article selection. Abbreviations: ANC/PNC, antenatal and postnatal care.

All identified references were uploaded to Rayyan [28]. We extracted the DOI number, year of publication, authors, title and abstract for each reference. Initially, one reviewer (AR) conducted a title‐and‐abstract screening for all articles identified through the search strategy to de‐duplicate articles. Subsequently, one reviewer (AR) independently evaluated the titles and abstracts to determine if they met the eligibility criteria. To assess the reliability of the screening process, a second reviewer (ZE) screened the titles and abstracts of a random 10% of all articles, resulting in a 94% agreement. Discrepancies between the reviewers were resolved through consensus or by consulting a third reviewer (DJD/BH). Articles that met the eligibility criteria were then obtained in full‐text and reviewed by the same two reviewers (AR/ZE), and conflicts were resolved through consensus. The included articles were presented and discussed within the research team for final approval. Reasons for exclusion were recorded accordingly (Figure 1).

### 2.3 Data extraction and management

We used a standardized data extraction form to extract study design attributes and methodologies along with baseline characteristics (e.g. study population, sample size, median gestational age) and essential outcomes of interest (i.e. PrEP initiation, continuation and adherence). Definitions used for each outcome of interest were also extracted. The heterogeneity in study design, objectives, populations included, outcome definitions and follow‐up periods among the included articles precluded a meta‐analysis.

### 2.4 Risk of bias assessment

We used the modified Newcastle Ottawa scale and Cochrane Risk of Bias 2 (ROB2) tools to assess the risk of bias of observational and experimental studies, respectively. The Newcastle Ottawa scale evaluates biases such as representativeness of the cohort, ascertainment of exposure and assessment of outcome. The ROB2 tool examines randomization processes, deviations from intended interventions and measurement of outcomes. This assessment helped identify factors in study design, conduct or reporting that may skew results, affecting their validity. Two reviewers (AR and ZE) independently evaluated the risk of bias in each study, and any discrepancies were resolved through mutual agreement or by consulting a third reviewer (BH/DJD). Studies were not excluded based on their risk of bias score.

## 3 RESULTS

### 3.1 Description of included studies

The search yielded 481 unique records, of which 30 were reviewed as full text and 12 met all eligibility criteria (Figure 1). The secondary search of references did not identify any additional records.

The 12 records report results from six parent studies conducted in four countries, that is Kenya (*n* = 5) [17, 18, 29, 30, 31], South Africa (*n* = 5) [16, 32, 33, 34, 35], Lesotho (*n* = 1) [36] and Malawi (*n* = 1) [37] (Table 1). Six of the studies were cohort studies [16, 17, 18, 30, 33, 34], four were (cluster) randomized controlled trials (RCTs) [29, 32, 35, 37], one was a pre‐/post‐evaluation [31] and one was a retrospective chart review [36]. All studies were conducted in public health clinics, three also recruited through private‐sector maternal and child health clinics in Kenya [17, 30, 31]. Sample sizes ranged from 106 to 9376 participants, with follow‐up periods varying from 1 month post‐enrolment to 12 months postpartum. Refill durations for PrEP ranged from 1 to 3 months of supply. All studies enrolled pregnant women; three studies also enrolled postpartum women [17, 30, 31, 32, 36]. Median gestational age at enrolment ranged from 20 to 26 weeks. The study objectives included: describing PrEP initiation (*n* = 7), continuation (*n* = 10) and adherence (either self‐reported or objective, *n* = 10), as well as evaluating the effect of an intervention on one or more PrEP use outcomes (*n* = 5). Among the five intervention studies, two focused on enhancing PrEP continuation and adherence; a Malawian pilot trial evaluating a combination package, and a Kenyan pre‐/post study investigating a two‐way SMS intervention [31, 37]. Two studies aimed to improve all three outcomes; a South African RCT assessing sexually transmitted infection (STI) point‐of‐care testing (i.e. *Chlamydia trachomatis*, *Neisseria gonorhoeae* and *Trichomonas vaginalis*), and a Kenyan cluster RCT comparing universal versus risk‐based PrEP delivery [29, 35]. A South African RCT implemented partner HIV self‐testing and real‐time biofeedback to enhance adherence [32].

**Table 1:** Characteristics of articles eligible for inclusion (*N*=12)

Study, first author, publication year	Country and study period	Study design	Study objectives	Study population	Type of clinic	Sample size	Median gestational age	Follow‐up time
Chi, 2024 [37]	Malawi, June 2020–November 2020	Randomized pilot trial	Evaluate the effectiveness of a combination package (patient‐centred counselling and the option of a participant‐selected adherence supporter) (intervention) to enhance antenatal and postnatal PrEP use versus SOC	Pregnant women, aged ≥ 18 years	Public ANC, Lilongwe	200; 100 intervention, 100 control	26 weeks (IQR 19–33)	6 months post‐enrolment
Masenyetse, 2023 [36]	Lesotho, January 2019–May 2022	Retrospective chart review	Characterize the PrEP cascade and use patterns among pregnant and postpartum women	Pregnant and postpartum women^a^ screened for PrEP and/or enrolled in PrEP programmes (January 2019–June 2021)	26 government/Christian Health Association‐run healthcare facilities, Lesotho	389	NR	NA
**PrEP in Pregnant and Postpartum women (PrEP‐PP) in South Africa**								
Joseph Davey, 2022 [16]	August 2019–October 2021	Prospective cohort	Evaluate PrEP initiation and continuation, and correlates of these outcomes	Pregnant adolescent girls and women, aged ≥ 16 years	Public health clinic, Cape Town	1201	21 weeks (IQR 15–31)	12 months postpartum
Davey, 2021 [32]	August 2020–April 2021	Randomized controlled pilot trial	Test impact of a combined intervention (HIVST for PrEP users and male partners, and real‐time adherence biofeedback) (intervention) on PrEP adherence versus SOC	Postpartum cisgender adolescent girls and women (4–24 weeks postpartum), aged ≥ 16 years, initiating PrEP		106; 53 intervention; 53 control	NA	1 month post‐enrolment
Joseph Davey, 2022 [33]	August 2019–October 2021	Prospective cohort	Quantify PrEP use by measuring TFV‐DP in DBS in a cohort study	Pregnant adolescent girls and women, aged ≥ 16 years, reported PrEP use in the last 30 days		382	54% (206/382) ≥ 20 weeks	12 months postpartum
Khadka, 2023 [34]	August 2019–October 2021	Prospective cohort	Evaluate PrEP initiation, continuation and persistence	Pregnant and postpartum AGYW, aged 16–24 years		486	24 weeks (IQR 17–34)	12 months postpartum
**Sexually Transmitted Infections and PrEP in Pregnancy Study (STIPPS)**								
De Voux, 2023 [35]	South Africa, November 2021–May 2022	Randomized controlled trial	Evaluate the effect of STI POC testing (intervention) on PrEP initiation, early persistence and adherence versus SOC	Pregnant women ≤ 34 weeks, aged ≥ 18 years	Public antenatal clinic, Cape Town	268; 133 intervention; 135 control	22 weeks (IQR 18–26)	1 month post‐enrolment
**PrEP Implementation for Mothers in Antenatal care (PrIMA) in Kenya**								
Kinuthia, 2023 [29]	January 2018–July 2021	Cluster randomized trial	Compare universal (intervention) and targeted (risk‐guided) approaches to PrEP delivery	Pregnant adolescent girls and women, aged ≥ 15 years	20 public ANCs, Siaya and Homa Bay counties	4447; 2250 intervention; 2197 control	24 weeks (IQR 20–30)	9 months postpartum
Pintye, 2023 [18]	January 2018–July 2021	Prospective cohort	Quantify and identify cofactors of PrEP initiation, persistence and adherence			2949		
**PrEP Implementation for Young women and Adolescents (PriYA) in Kenya**								
Kinuthia, 2020 [17]	November 2017–December 2018	Prospective cohort	Provide real‐world evidence on delivering PrEP in maternal and child health clinics	Adolescent girls and women, aged >15 years, receiving antenatal care or child welfare services	16 public, faith‐based and private sector maternal and child health clinics, Kisumu County	9376; 4912 pregnant; 4464 postpartum	26 weeks (IQR 20–32)	6 months post‐initiation
Pintye, 2020 [30]	November 2017–December 2018	Prospective cohort	Evaluate detection of TFV‐DP among women who initiated PrEP			233 DBS samples from 201 unique women^b^	NR	NA
Pintye, 2020 [31]	February 2018–October 2018	Pre‐/post‐evaluation	Assess implementation of a two‐way SMS intervention on PrEP continuation and adherence			190^c^	25 weeks (IQR 20–28)	1 month post‐enrolment

Abbreviations: AGYW, adolescent girls and young women; ANC, antenatal care; DBS, dried blood spot; HIVST, HIV self‐testing; IQR, interquartile range; NA, not applicable; NR, not reported; PNC, postnatal care; POC, point‐of‐care; PrEP, pre‐exposure prophylaxis; SOC, standard of care; SMS, short message service; STI, sexually transmitted infection; TFV‐DP, tenofovir‐diphosphate.

^a^Those pregnant and postpartum women with a documented entry point through antenatal or postnatal care service points.

^b^Attending first PrEP follow‐up visit at a median of 5 weeks of PrEP initiation.

^c^Enrolled in intervention.

### 3.2 Risk of bias

The Newcastle Ottawa scale identified potential bias in three studies due to unaccounted loss‐to‐follow‐up (Table 2). The ROB‐2 assessment revealed some bias in all (quasi)experimental studies, mainly due to a lack of blinding and uncertainty regarding the presence of a statistical analysis plan (Table 2). Notably, the inability to blind participants and providers was inherent to the nature of the study interventions, such as the use of an SMS communication platform. The Kenyan PrEP implementation for young women and adolescents (PriYA) study had a high risk of bias due to its pre‐post design and missing outcome data, as 60% (99/166) of women in the control arm and 46% (87/190) in the intervention arm were lost to follow‐up at 1 month [31]. Concerns were also raised about the absence of an objective adherence measurement, also noted in the Kenyan PrEP Implementation for Mothers in Antenatal care (PrIMA) study [29, 31].

**Table 2:** Risk of bias assessment

Observational studies						
Newcastle‐Ottawa Scale	Representativeness of the cohort	Ascertainment of exposure	Assessment of outcome	Adequate follow‐up duration	Loss‐to follow‐up accounted for	Total
PrEP in Pregnant and Postpartum women (PrEP‐PP) in South Africa						
Joseph Davey et al., 2022 [16]	1	1	1	1	0	4
Joseph Davey et al., 2022 [33]	1	1	1	1	1	5
Khadka et al., 2023 [34]	1	1	1	1	1	4
PrEP Implementation for Mothers in Antenatal care (PrIMA) in Kenya						
Pintye et al., 2023 [18]	1	1	1	1	1	5
PrEP Implementation for Young women and Adolescents (PriYA) in Kenya						
Kinuthia et al., 2020 [17]	1	1	1	1	0	4
Pintye et al., 2020 [30]	1	1	1	1	1	5
Masenyetse et al., Lesotho, 2023 [36]	1	1	1	1	0	4

(Quasi) experimental studies						
Risk of Bias 2 score domains	Randomization process	Deviations from intended interventions	Missing outcome data	Measurement of the outcome	Selection of the reported result	Overall
Chi et al., Malawi, 2024 [37]	Low risk	Some concerns	Low risk	Low risk	Some concerns	Some concerns
PrEP in Pregnant and Postpartum women (PrEP‐PP) in South Africa (sub‐study on HIVST + urine TFV biofeedback)						
Davey et al., 2021 [32]	Low risk	Some concerns	Low risk	Low risk	Some concerns	Some concerns
Sexually Transmitted Infections and PrEP in Pregnancy Study (STIPPS) in South Africa						
De Voux et al., 2023 [35]	Low risk	Some concerns	Low risk	Low risk	Some concerns	Some concerns
PrEP Implementation for Mothers in Antenatal care (PrIMA) in Kenya						
Kinuthia et al., 2023 [29]	Low risk	Some concerns	Low risk	Some concerns	Some concerns	Some concerns
PrEP Implementation for Young women and Adolescents (PriYA) in Kenya						
Pintye et al., 2020 [31]	High risk	Some concerns	Some concerns	Some concerns	Some concerns	Some concerns

*Note*: The Modified Newcastle‐Ottawa scale assigns stars based on the quality of each domain, with a maximum score of 5 indicating a well‐designed study with minimal or no bias, and a score of 0 representing a flawed study with significant bias across all domains.

Abbreviations: HIVST, HIV self‐testing; TFV, tenofovir.

### 3.3 PrEP initiation

Seven studies reported on PrEP initiation, defined as the percentage of women either accepting a PrEP prescription at the first or any ANC/PNC visit or self‐reporting PrEP use at a follow‐up visit after accepting PrEP at a prior ANC/PNC visit, among those eligible for PrEP (Table 3).

**Table 3:** Studies reporting on PrEP initiation and continuation

		Initiation		Continuation post‐enrolment			
					1 month	3 months	6 months (unless otherwise reported)
Study reference	Study population	Definition^a^	% (*n*/*N*)	Definition	% (*n*/*N*)	% (*n*/*N*)	% (*n*/*N*)
**Observational studies**							
**PrEP in Pregnant and Postpartum women (PrEP‐PP) in South Africa**							
Joseph Davey et al., 2022 [16]	Pregnant adolescent girls and women, aged ≥ 16 years	Accepting PrEP prescription at first ANC visit (baseline)	84% (1014/1201)	Receiving a PrEP prescription at both baseline and the 3‐month follow‐up visit	66% (629/953)^b^	58% (493/844)^c^	NR
Khadka et al., 2023 [34]	Pregnant and postpartum AGYW, aged 16–24 years		83% (403/486)	Receiving a PrEP prescription at each study visit after the baseline visit among those who initiated PrEP at baseline	63% (253/403)	54% (212/395)	39% (149/380)
**PrEP Implementation for Mothers in Antenatal care (PrIMA)**							
Pintye et al., Kenya, 2023 [18]	Pregnant adolescent girls and women aged ≥ 15 years	Participant reports swallowing PrEP pills at a follow‐up visit	2949 were offered PrEP, 14% (405/2949) initiated	Participant reports continuing with PrEP medication	NR	NR	58% (212/363)^3^, ^4^
**PrEP Implementation for Young women and Adolescents (PriYA)**							
Kinuthia et al., Kenya, 2020 [17]	Adolescent girls and women, aged >15 years, receiving antenatal care or child welfare services	NR	9376 women were offered PrEP, 22% (2030/9376) initiated	NR	39% (786/2030)	22% (441/2030)	12% (189/1618)
Masenyetse et al., Lesotho, 2023 [36]	Pregnant and postpartum women screened for PrEP and/or enrolled in PrEP programmes (January 2019–June 2021)	NA^f^	389 pregnant or postpartum women identified from ANC/PNC service points initiating on PrEP	Participants having any documented PrEP FU visit after PrEP initiation	40% (156/389) no recorded FU visits; 20% (76/389) has one recorded PrEP FU visit; 40% (157/389) had at least two documented PrEP FU visits	NR	NR
**(Quasi) experimental studies**							
Chi et al., Malawi, 2024 [37]	Pregnant women, aged ≥ 18 years	NA	I: 100 C: 100	Participants presenting for study visits were classified as retained	I: 94% (94/100) C: 92% (92/100)	I: 90% (90/100) C: 89% (89/100)	I: 88% (88/100) C: 83% (83/100)
**PrEP in Pregnant and Postpartum women (PrEP‐PP)** (sub‐study on HIVST + urine TFV biofeedback)							
Davey et al., South Africa, 2021 [32]	Postpartum cisgender adolescent girls and women, aged ≥ 16 years, initiated PrEP during recent pregnancy	NA	I: 53 C: 53	NA	I: 91% (48/53) C: 98% (52/53)	NR	NR
**Sexually Transmitted Infections and PrEP in Pregnancy Study (STIPPS)**							
De Voux et al., South Africa, 2023 [35]	Pregnant women ≤ 34 weeks, aged ≥ 18 years	Number and proportion of participants who initiated PrEP at baseline	I: 67% (89/133) C: 62% (84/135)	Proportion of participants who initiated PrEP and returned to the clinic to refill their PrEP prescription	I: 79% (70/133) C: 80% (67/135)	NR	NR
**PrEP Implementation for Mothers in Antenatal care (PrIMA)**							
Kinuthia et al., Kenya, 2023 [29]	Pregnant adolescent girls and women, aged ≥ 15 years	PrEP acceptance: participant accepted PrEP at any visit; PrEP confirmation visit: participant reports swallowing PrEP pills at visits after PrEP acceptance with the PrEP initiation date defined as the median date between the PrEP acceptance and PrEP confirmation visits.	I: 64% (2250/3537) enrolled; 20% (441/2250) accepted PrEP; 18% (397/2250) initiated PrEP (universal) C: 45% (2197/4890) enrolled, 18% (387/2197) accepted, 15% (323/2197) initiated (targeted)	PrEP duration: time between the PrEP initiation date and discontinuation or study end.	NR	NR	I: Median: 8.6 (IQR 3.2–11.4) months in FU^g^ C: Median: 9.0 (IQR 3.8–11.9) months in FU^g^
**PrEP Implementation for Young women and Adolescents (PriYA)**							
Pintye et al., Kenya, 2020 [31]	Adolescent girls and women, aged >15 years	NA	I: 190 C: 166	A confirmed dispensation of a PrEP refill at an attended follow‐up visit	I: 43% (81/190) C: 22% (37/166)	NR	NR

Abbreviations: AGYW, adolescent girls and young women; ANC, antenatal care; FU, follow‐up; HIVST, HIV self‐testing; NA, not applicable; NR, not reported; PNC, postnatal care; PrEP, pre‐exposure prophylaxis; TFV, tenofovir.

^a^Among participants eligible for PrEP.

^b^ *N* = 61 women were censored at 1 month (e.g. due to pregnancy loss).

^c^At the timing of interim analysis, 844 women were eligible for their 3‐month PrEP refill.

^d^ *N* = 42 women restarted PrEP after stopping and were excluded from continuation analysis.

^e^Follow‐up period was defined as 9 months postpartum.

^f^No records of pregnant or postpartum women being screened and not initiating PrEP.

^g^PrEP duration in months, to the first reported discontinuation of PrEP or study end.

In the observational studies (*n* = 5), initiation ranged from 14% to 84% (Table 3). The initiation of 84% was reported in the PrEP‐PP cohort study in South Africa, where 1014 out of 1201 pregnant adolescent girls and women aged ≥ 16 accepted a PrEP prescription at their first ANC visit [16]. PrEP initiation was higher among those diagnosed and treated for an STI (93%, 153/165) compared to those not diagnosed with an STI (84%, 707/845; *p* < 0.001) [16]. A sub‐analysis of the PrEP‐PP study found a similar initiation of 83% (403/486) among pregnant adolescent girls and young women (AGYW) aged 16–24 [34].

In contrast, the PriMA cohort sub‐study in Kenya reported an initiation (defined as the participant‐reported swallowing of PrEP pills following acceptance at a prior visit) of 14% (405/2949) among adolescent girls and women aged ≥ 15 in their second trimester [18]. Women were more likely to initiate PrEP if they had a syphilis diagnosis during pregnancy (adjusted RR [aRR]: 1.84, 95% CI 1.24–2.75, *p* = 0.003) or a partner known to be living with HIV (aRR: 6.37, 95% CI 4.18–0.69, *p* < 0.001) [18]. The PriYA cohort study in Kenya reported that 22% (2030/9376) of pregnant or postpartum adolescent girls and women aged > 15 receiving ANC or child welfare services initiated PrEP, though specific definitions of initiation were not provided [17]. Uptake was highest among women whose partners were living with HIV (79%, 153/193) (adjusted prevalence ratio: 6.96, 95% CI 5.46–8.89, *p* < 0.001) compared to women with partners of unknown HIV status (37%, 1178/3165) (adjusted prevalence ratio: 3.08, 95% CI 2.50–3.81, *p* < 0.001), and partners not living with HIV (12%, 696/5997) [17]. Women who were diagnosed or treated for an STI were also more likely to initiate PrEP (adjusted prevalence ratio: 1.57, 95% CI 1.20–2.06, *p* < 0.001).

In the experimental studies (*n* = 2), PrEP initiation ranged from 15% to 67% (Table 3). In the PrIMA trial in Kenya, 20 public maternal and child health clinics were randomized to universal (i.e. all women received PrEP counselling) or targeted (i.e. HIV risk‐guided) PrEP counselling/offer [29]. Initiation in the universal arm was 18% (397/2250) compared to 15% (323/2197) in the targeted arm (aRR: 0.68, 95% CI 0.46–1.02, *p* = 0.062) [29]. There was no significant difference in appropriate PrEP use decision‐making, meaning, PrEP initiation among those at high risk of acquiring HIV and no PrEP initiation for those not at risk, between the universal (68%) versus targeted arm (59%) (aRR = 1.03, 95% CI = 0.96–1.10, *p* = 0.37) [29]. In the RCT of STI screening and PrEP in pregnancy study (STIPPS) in South Africa, pregnant women were randomized to either point‐of‐care STI screening (i.e. *Chlamydia trachomatis*, *Neisseria gonorrhoea* and *Trichomonas vaginalis* using Cepheid GeneXpert) versus syndromic management [35]. There was no difference in PrEP initiation in the point‐of‐care arm (67%; 89/133) compared to syndromic management (62% [84/135], *p* = 0.42); however, an STI diagnosis (i.e. positive STI test or reporting STI symptoms) at initiation was associated with higher PrEP initiation (aRR = 1.28, 95% CI = 1.08–1.52, *p* = 0.00), when controlling for study arm, maternal and gestational age [35].

### 3.4 Continuation

Ten studies reported on PrEP continuation or persistence, with the definitions used for each outcome detailed in Table 3. In the South African PrEP‐PP study, 66% (629/953) of pregnant women who initiated PrEP received a prescription at 1 month and 58% (493/844) at 3 months follow‐up, indicating continued use at 3 months post‐enrolment [16]. Slightly lower persistence was reported in the sub‐analysis among pregnant and postpartum AGYW (1‐month follow‐up: 63% [253/403], 3 months: 54% [212/395]) [34].

In the Kenyan PrIMA study, which defined continuation based on self‐reported PrEP use, 58% (212/363) of initiators continued PrEP use at 9 months postpartum, excluding 42 women who had restarted PrEP after stopping [18]. The lowest continuation was observed in the Kenyan PriYA study, which declined from 39% at 1 month to 12% at 6 months [17]. Data from Lesotho demonstrated that 40% (157/389) of women had at least two PrEP follow‐up visits after initiating PrEP, while an equal proportion (40%, 156/389) had no follow‐up visits [36].

In a pilot RCT in Malawi, which aimed to evaluate the effect of a combination intervention package, including integrated Next Step Counselling and an adherence supporter option on retention and adherence compared to standard care [37], continuation was 90% (90/100) versus 89% (89/100), respectively, at 3 months (adjusted probability difference [aPD]: 5.1%, 95% CI –3.7%, 14.0%) and 88% versus 83%, respectively, at 6 months (aPD: 7.4%, 95% CI –2.7%, 17.4%) [37]. A pilot RCT in South Africa, embedded in the PrEP‐PP study, offered postpartum women adherence biofeedback counselling after point‐of‐care urine tenofovir testing and HIV self‐testing for them and their partners [32]. The control group received standard‐of‐care routine counselling without biofeedback and facility‐based HIV tests [32]. At 1 month post‐enrolment, 91% (48/53) of women in the intervention arm and 98% (52/53) in the control arm returned for PrEP [32]. The PrIMA cluster randomized trial in Kenya reported no differences between the intervention and control arms in terms of duration of PrEP use. They found that the median duration of PrEP use did not differ between arms (9 months in the targeted arm and 8.6 months in the universal arm) [29].

A pre‐/post‐evaluation sub‐study embedded in the Kenyan PriYA study offered a two‐way SMS communication platform to pregnant and postpartum women [31]. Compared to women who initiated PrEP the month before implementation, women who received SMS reminders were more likely to return for their first PrEP follow‐up visit (40% [67/166] vs. 53% [101/190], aRR: 1.26, 95% CI 1.06–1.50, *p* = 0.008) and more likely to continue with PrEP (22%, 37/166 vs. 43%, 81/190, aRR: 1.75, 95% CI 1.21–2.55, *p* = 0.003) [31]. In the STIPPS RCT in South Africa, 79% (70/133) of women in the intervention arm (i.e. point‐of‐care STI testing) and 80% (67/135) in the control arm (i.e. syndromic STI management) returned at 1‐month visit and requested a PrEP prescription (*p* = 0.49) [35].

### 3.5 Adherence

Ten studies—five observational, four experimental and one quasi‐experimental—reported on PrEP adherence; three measured self‐reported adherence only [16, 29, 31] defined as the number of missed doses or daily use in the past 30 days (Table 4). Three studies measured adherence objectively [30, 34, 37] through tenofovir‐diphosphate (TFV‐DP) concentrations in dried blood spots (DBS), tenofovir plasma concentrations from women who reported PrEP use in the past 30 days or through tenofovir detection in urine.

**Table 4:** Studies reporting on self‐reported and/or objective PrEP adherence

		Definition		Adherence post‐enrolment (unless otherwise reported)					
				1 month		3 months		≥ 6 months	
Study reference	Study population	Self‐reported	Objective	Self‐reported	Objective	Self‐reported	Objective	Self‐reported	Objective
**Observational studies**									
**PrEP in Pregnant and Postpartum women (PrEP‐PP) in South Africa**									
Joseph Davey et al., 2022 [16]	Pregnant adolescent girls and women, aged ≥ 16 years	Missed daily doses in the past month	NA	NR	NR	46% (387/844) missed ≥ 1 daily doses	NR	NR	NR
Joseph Davey et al., 2022 [33]	Pregnant adolescent girls and women, aged ≥ 16 years, reported PrEP use in the last 30 days	Self‐reported PrEP use in the last 30 days before study visit	TFV‐DP in DBS in women who reported taking PrEP in the last 30 days prior to the visit. Lower limit of quantification for TFV‐DP: 16.6 fmol/3‐mm punch. Separate thresholds for pregnant versus postpartum women	NR	NR	**pregnant women**: 81% (148/183) and **postpartum women**: 71% (97/136) reported daily use 63 women did not report PrEP use in the past 30 days	**pregnant women**: 67% (122/183): any TFV‐DP detected, of those 37% (68/183) TFV‐DP ≥ 2 doses/week, 7% (14/183) TFV‐DP 7 doses/week **postpartum women**: 60% (82/136): any TFV‐DP detected, of those 31% (42/136) TFV‐DP ≥ 2 doses/week, 2% (3/136) TFV‐DP 7 doses/week	**pregnant women**: 79% (46/58) and **postpartum women**: 76% (89/117) reported daily use	**pregnant women**: 29% (17/58) and **postpartum women**: 22% (26/117) TFV‐DP ≥ 2 doses/week
Khadka et al., 2023 [34]	Pregnant and postpartum AGYW, aged 16–24 years	NA	TFV‐DP in DBS in women who reported taking PrEP in the last 30 days prior to the visit. Separate thresholds for adherence in pregnant versus postpartum women.	NR	NR	NR	TFV‐DP was detected for 49% (85/175); 51% (90/175) had unquantifiable levels or did not report PrEP use in the last 30 days; 23% (40/175): < 2 doses/week; 20% (35/175): 2–5 doses/week; 6% (10/175): 7 doses/week	NR	TFV‐DP detected for 20% (21/107); 80% (80/107) had unquantifiable levels or did not report PrEP use in the last 30 days; 14% (15/107): < 2 doses/week; 5% (5/107): 2–5 doses/week; 1% (1/107): 7 doses/week
**PrEP Implementation for Mothers in Antenatal care (PrIMA)**									
Pintye et al., Kenya, 2023 [18]	Pregnant adolescent girls and women aged ≥ 15 years	Number of missed doses in the past 30 days	TFV‐DP in DBS from visits with self‐reported PrEP use in the past 30 days. Lower limit of quantification for TFV‐DP: 25 fmol/punch	NR	NR	NR	NR	at 9 months postpartum: 54% (114/212) not missing any PrEP pills in the last 30 days at 9 months postpartum	Among DBS randomly selected from visits where participants continued with PrEP, 50% (214/427) had quantifiable TFV‐DP (**pregnant women: 53% vs. postpartum: 27%)**; 26% (111/427): TFV‐DP < 2 doses/week; 65% (278/427): TFV‐DP 2–6 doses/week; 9% (38/427): TFV‐DP 7 doses/week.
**PrEP Implementation for Young women and Adolescents (PriYA)**									
Pintye et al., Kenya, 2020 [30]	Adolescent girls and women, aged >15 years, receiving antenatal care or child welfare services	NA	TFV‐DP in DBS from first follow‐up visit scheduled 30 days post‐PrEP initiation among participants reporting using PrEP in the last 14 days. Lower limit of quantification for TFV‐DP: 25 fmol/punch	NR	62% (125/201) of DBS samples had detectable TFV‐DP; median 492 fmol/sample (IQR 335–718); 30% (61/201) had >500 fmol/sample (≥ 4 doses/week)^a^	NR	NR	NR	Among DBS from first FU visits: 62% (125/201) had detectable TFV‐DP, median concentration 492 fmol/sample (IQR 335–718); **postpartum women**: 66% (100/152) versus **pregnant women**: 51% (25/49). Among DBS from later FU visits: 90% (28/31) DBS samples had quantifiable TFV‐DP, median concentration 635 fmol/sample (IQR 436–761).^b^
**(Quasi) experimental studies**									
Chi et al., Malawi, 2024 [37]	Pregnant women, ≥ 18 years	NA	Plasma TFV (days) and intracellular TFV‐DP in upper layer packed cells and scored according to algorithm by Corneli et al.	NR	NR	NR	**I**: 31% (28/89) and **C**: 33% (29/89) had 4–7 doses/week	NR	**I**: 25% (22/88) and **C**: 32% (26/82): had 4–7 doses/week
**PrEP in Pregnant and Postpartum women (PrEP‐PP)** (sub‐study on HIVST + urine TFV biofeedback)									
Davey et al., South Africa, 2021 [32]	Postpartum cisgender adolescent girls and women (4–24 weeks postpartum), aged ≥ 16 years, initiated PrEP during recent pregnancy	Self‐reported PrEP use in the past week	POC TFV detection in urine (reflecting adherence in past 48–72 hours)	**I**: 77% (41/53) and **C**: 81% (43/53) reported daily use in past week	**I**: 62% (33/53) and **C**: 34% (18/53) had quantifiable TFV in urine	NR	NR	NR	NR
**Sexually Transmitted Infections and PrEP in Pregnancy Study (STIPPS)**									
De Voux et al., South Africa, 2023 [35]	Pregnant women ≤ 34 weeks, ≥ 18 years	Number of pills taken in the past week	TFV‐DP in DBS among participants who initiated PrEP at baseline	**I**: 11% (10/89) and **C**: 12% (10/84) reported daily use in the past 7 days	**I**: 49% (44/89) and **C**: 45% (38/84) had detectable TFV‐DP	NR	NR	NR	NR
**PrEP Implementation for Mothers in Antenatal care (PrIMA)**									
Kinuthia et al., Kenya, 2023 [29]	Pregnant adolescent girls and women, aged ≥ 15 years	Participant‐report of swallowing PrEP pills; dichotomized as no missed doses versus any missed doses in the past 30 days	NA	**I**: 53% (206/2250) and **C**: 63% (200/2197) daily use in the past 30 days prior PrEP confirmation visit^c^	NR	NR	NR	NR	NR
**PrEP Implementation for Young women and Adolescents (PriYA)**									
Pintye et al., Kenya, 2020 [31]	Adolescent girls and women, aged >15 years, receiving antenatal care or child welfare services	Number of missed PrEP doses in the past month	NA	**I**: 73% (74/101) and **C**: 55% (37/67) < 1 missed pill/week	NR	NR	NR	NR	NR

Abbreviations: AGYW, adolescent girls and young women; ANC, antenatal care; c, control; DBS, dried blood spot; FU, follow‐up; HIVST, HIV self‐testing; I, intervention; IQR, interquartile range; NA, not applicable; NR, not reported; POC, point‐of‐care; PrEP, pre‐exposure prophylaxis; TFV, tenofovir; TFV‐DP, tenofovir‐diphosphate.

^a^Samples taken from unique women attending their first PrEP follow‐up visit, at a median of 5 weeks (IQR 4–18).

^b^Samples were from subsequent visits after first PrEP follow‐up visit among the same women, at a median of 24 weeks since PrEP initiation (IQR 17–37).

^c^A PrEP confirmation visit was defined as participant's first study visit after accepting PrEP.

In four studies that reported both measures, self‐reported PrEP use was higher than objective measures [18, 32, 33, 35]. In the South African PrEP‐PP study, among a subset of women returning for follow‐up and reporting PrEP use in the past 30 days, 81% (148/183) of pregnant women and 71% (97/136) of postpartum women reported daily PrEP use at 3 months [33]. TFV‐DP levels, corresponding to seven doses/week, were identified among 7% (14/183) of pregnant and 2% (3/136) of postpartum women [33]. At 6 months, self‐reported adherence was 79% (46/58) among pregnant and 76% (89/117) among postpartum women; objective adherence (TFV‐DP levels corresponding to ≥ 2 doses/week) was 29% (18/62) and 22% (25/113), respectively [33].

In the South African pilot RCT among postpartum women, 62% (33/53) of women in the intervention group (i.e. HIV biofeedback counselling after point‐of‐care tenofovir test and HIV self‐testing for them and their partners) had tenofovir in their urine at 1 month post‐enrolment compared to 34% (18/53) in the standard‐of‐care arm (risk ratio [RR] = 1.83, 95% CI = 1.19–2.82, *p* = 0.001) [32]. Discrepant results between self‐reported adherence and urine tenofovir tests were significantly lower in the intervention compared to the control arm (RR = 0.33; 95% CI 0.17–0.67, *p* = 0.03) [32].

The Kenyan PrIMA study found that 54% (114/212) of women reported no missed PrEP doses at 9 months postpartum. Among DBS collected at randomly selected visits where participants persisted with PrEP, 9% (38/427) had TFV‐DP levels reflecting daily use. Quantifiable TFV‐DP was twice as likely in pregnancy than postpartum (53% vs. 27%, aRR = 1.90; 95% CI = 1.40–2.57; *p* < 0.001) [18]. In the South African STIPPS study, 49% (44/89) of women in the point‐of‐care STI testing arm and 45% (38/84) in the syndromic management arm had detectable TFV‐DP in DBS at 1 month (*p* = 0.67) [35]. Self‐reported daily use in the past 7 days was reported by 11% (10/89) and 12% (10/84) of women, respectively (*p* = 0.69); no women had TFV‐DP levels equivalent to seven doses in the past week.

In the South African PrEP‐PP study, 46% (387/844) of pregnant women reported missing ≥ 1 dose in the last 30 days at 3 months post‐enrolment [16]. In the Kenyan PrIMA cluster randomized trial, 53% (206/2250) of women self‐reported PrEP use in the past 30 days at first study visit after accepting PrEP compared to 63% (200/2197) in the targeted arm (aRR: 1.13, 95% CI 0.91–1.41) [29]. In the Kenyan pre‐/post‐evaluation study, 73% (74/101) of women who returned for a follow‐up visit in the SMS intervention arm self‐reported PrEP adherence (i.e. < 1 missed pill/week) compared to 55% (37/67) in the control arm (aRR: 1.35; 95% CI 1.28–1.41; *p* < 0.001) [31].

In the Kenyan PriYA study, 62% (125/201) of DBS samples collected at a first follow‐up visit scheduled 30 days post‐PrEP initiation among participants who self‐reported having used PrEP in the last 14 days had quantifiable levels of TFV‐DP. Detectable TFV‐DP was more likely among postpartum women compared to pregnant women (66% [100/152] vs. 51% [25/49], RR: 1.29; *p* = 0.055) [30]. In the Malawian RCT, adherence consistent with 4–7 doses/week among intervention and control groups was low at 3 (31% vs. 33%, respectively) and 6 months (25% vs. 32%, respectively), with no significant differences observed between groups at 3 (aPD: −1.8%, 95% CI −16.2%, 12.7%) or 6 months (aPD: −5.5%, 95% CI −18.0%, 6.9%) [37]. In the PrEP‐PP sub‐study among pregnant AGYW, 49% (85/175) had detectable TFV‐DP at 3 months, dropping to 20% (21/107) at 6 months [34].

## 4 DISCUSSION

This systematic review is the first to comprehensively describe PrEP use—including initiation, continuation and adherence—among pregnant and postpartum women receiving ANC and/or PNC services. The integration of PrEP into these services demonstrated varying levels of PrEP initiation (from 14% to 84%). PrEP initiation was higher among women at increased risk of HIV acquisition, such as those diagnosed with an STI or with partners living with HIV. However, PrEP continuation and adherence declined rapidly in observational studies, especially during the postpartum period. While self‐reported adherence was generally high, objective adherence measurements revealed inadequate levels of adherence to effectively prevent HIV acquisition. Two‐way SMS communication and real‐time adherence biofeedback counselling using urine tenofovir testing enhanced PrEP continuation and adherence. These findings highlight that effective, sustained PrEP use among pregnant and postpartum women in ANC/PNC is suboptimal. Despite these challenges, the integration of PrEP into ANC/PNC services represents a promising, person‐centred approach to HIV prevention, providing HIV prevention support to pregnant and postpartum women during critical periods of vulnerability to HIV. Further interventions, tailored to the needs and preferences of these women, are necessary to enhance prevention‐effective PrEP use and maximize impact.

PrEP initiation among pregnant and postpartum women varied widely across studies. This variability could partially be explained by differing PrEP initiation definitions. Some studies measured initiation as prescription pick‐up at baseline [16, 33, 34], while others relied on self‐reported use during a follow‐up visit [18]. These varying definitions make direct comparisons of PrEP initiation challenging. The WHO and the European Centre for Disease Prevention and Control (ECDC) list PrEP uptake as a core indicator for monitoring and evaluating PrEP use, but allow flexibility in data source selection (e.g. written or filled prescription data, facility registers for self‐reported PrEP use) depending on local availability and context‐specific feasibility [38, 39]. While these PrEP use indicators, first published in 2018, provide a useful foundation, they may not fully reflect the context of pregnancy and the postpartum period. Revisiting these indicators could improve consistency in outcome measurement and better guide future research in this population. Additionally, while UNAIDS 2025 targets aim for 95% coverage of pregnant and breastfeeding women, among other key populations, with people‐centred HIV prevention programmes, no explicit PrEP coverage target exists for pregnant and postpartum women. Establishing such a target could enhance accountability and stimulate progress towards improved PrEP uptake among this population.

PrEP initiation was higher among women who were more likely to be exposed to HIV, such as those diagnosed with an STI or with partners living with HIV [16, 17, 18, 35]. This suggests that women's self‐perceived HIV acquisition risk and awareness of partner HIV status play critical roles in a woman's decision to start PrEP, as also reported in a qualitative study in Malawi on PrEP decision‐making among pregnant women [40]. However, targeted PrEP delivery based on risk scores resulted in the same levels of PrEP use and HIV incidence compared to delivery to all women [29], suggesting that universal PrEP delivery should be considered to reach pregnant and postpartum women in high HIV burden settings. Universal delivery could enable task‐shifting, facilitating group counselling strategies and broader PrEP education within maternal and child health services, thereby facilitating PrEP integration into ANC/PNC [29]. By informing all women receiving ANC/PNC about PrEP availability, following regular HIV testing, this approach may also promote person‐centred care, by addressing individuals’ needs in a non‐stigmatizing, inclusive manner, similar to the universal offer of provider‐initiated HIV testing and counselling [11]. It can empower women to make informed decisions about their health and increase community awareness [29].

The median gestational age at PrEP initiation was 20–26 weeks, as such, many women were not using PrEP prior to pregnancy (in the conception period) nor during their first trimester. Delayed initiation could result from late presentation to ANC (i.e. second or third trimester) as PrEP was mostly offered during women's first ANC visit. These findings underscore the need to develop and implement strategies to promote PrEP integration into family planning services for conception coverage and earlier ANC engagement and PrEP initiation to ensure timely use of prevention services [41]. Strategies might include community outreach, involving pharmacies, to increase awareness of ANC benefits and integrating PrEP education into pre‐pregnancy counselling, family planning services and STI care [41].

PrEP continuation declined rapidly within the first 3 months after initiation, particularly in the observational studies, with further declines in the postpartum period. Two‐way SMS communication enhanced early PrEP continuation, while patient‐centred counselling showed no effect, highlighting mixed effects of these interventions in promoting early PrEP continuation/adherence [31, 37]. In comparison, findings from an RCT among young Kenyan women in general, evaluating the effect of SMS reminders on PrEP adherence over a 2‐year period, showed that SMS reminders were ineffective in promoting PrEP continuation/adherence [42]. With mobile health (“mHealth”) interventions, a frequently investigated option to enhance effective PrEP use, awareness of the digital divide is warranted [43, 44]. For example, in the Kenyan two‐way SMS communication study, nearly half of the women screened for study enrolment were ineligible due to cell phone‐related issues (e.g. not having a phone). As such, the intervention excluded a large population who lacked access to the necessary technology to benefit from it; reflecting a substantial digital divide. This underscores the importance of thoughtfully leveraging digital technology by developing, with pregnant and postpartum women, and evaluating digital health interventions that are tailored to the specific needs of pregnant and postpartum women and are cognizant of the digital divide.

This review highlights that adherence to oral PrEP is a substantial challenge among pregnant and postpartum women. Discrepancies between self‐reported and objective adherence measures were common. While early adherence improved among participants receiving adherence biofeedback counselling after point‐of‐care urine tenofovir testing compared to standard‐of‐care [32], patient‐centred counselling was ineffective in another study [37]. Long‐acting PrEP formulations, such as twice‐yearly lenacapavir and bi‐monthly cabotegravir injections, the new 3‐monthly dapivirine vaginal ring and MK‐8527, a monthly PrEP pill, found to be safe and well‐tolerated in a phase II clinical trial, offering promising solutions to address adherence issues [22, 23, 24, 25, 45, 46]. A phase III efficacy study is planned for late 2025. A study in Kenya and South Africa reported that 75% of pregnant and postpartum women who had used oral PrEP expressed a theoretical preference for long‐acting injectable PrEP, suggesting its potential to improve effective use [47]. Similarly, pregnant and breastfeeding women participating in a discrete choice experiment in Botswana and South Africa strongly favoured long‐acting injectable PrEP over oral PrEP [48]. Evidence from family planning has shown that expanded choice improves product use, but there is less evidence of this effect for PrEP, which involves different challenges, such as recognizing the risk of acquiring HIV [49]. Understanding how pregnant and postpartum women make decisions about using these female‐controlled PrEP products, uptake, switching products and continuation in real‐life will be critical to inform person‐centred care to reduce HIV incidence among women and their children. Ongoing collection of safety data for these products during pregnancy and breastfeeding is essential to ensure their safe and effective use among these populations.

Postpartum PrEP continuation and adherence posed challenges. Studies suggest that this decrease is often driven by changes in risk perception, such as postpartum abstinence, or reduced perceived HIV risk, alongside less frequent contact with healthcare facilities during the postpartum period [17, 19]. As sexual activity often resumes after childbirth, and the risk of HIV transmission via breastfeeding remains substantial, the postpartum period is a critical time for HIV prevention [2, 3, 50]. To address these challenges, future strategies should also offer the option of decentralized, person‐centred models as being developed and scaled‐up among other key populations, such as men who have sex with men, for oral PrEP and for the delivery of antiretroviral therapy [51]. Specifically, efforts could focus on community‐based delivery approaches, which prioritize postpartum women, such as establishing community pick‐up points and mobile services, and engaging pharmacies, co‐created with postpartum women based on their needs and values, could enhance the accessibility and uptake of PrEP among these women [52].

This systematic review has limitations. This review focused on oral PrEP, excluding other prevention methods such as the vaginal ring and long‐acting injectable PrEP. Additionally, PrEP delivery was limited to ANC/PNC settings, which may have resulted in an underestimation of overall PrEP use among pregnant and postpartum women. Furthermore, we limited our search to English papers and excluded conference abstracts, which may have resulted in an underrepresentation of evidence from non‐English speaking countries and novel insights, potentially affecting the comprehensiveness and generalizability of our findings. Despite these limitations, the review, which involved multiple reviewers and searching multiple databases, highlights critical knowledge gaps and emphasizes the importance of further research on PrEP use in this vulnerable population. The findings contribute valuable insights into integrating PrEP into maternal health services in regions with high HIV prevalence.

Limitations may be present within the studies included in this review. The number of published and included studies on PrEP use among pregnant and postpartum women remained limited, with most studies conducted in Kenya and South Africa, limiting generalizability to other countries and regions. Definitions used for PrEP outcomes also varied across studies, limiting comparability between studies. The lack of long‐term follow‐up in many studies, often limited to 1–3 months, restricts understanding of long‐term PrEP continuation and adherence. More longitudinal research is needed to assess how PrEP continuation changes through pregnancy and postpartum periods. Despite quality control measures and expert input, the absence of a full dual‐reviewer screening may have limited the review's comprehensiveness. Moreover, several studies were conducted during the COVID‐19 pandemic, which may have influenced healthcare access and PrEP use, potentially skewing results. The potential bias in studies not reporting women's loss‐to‐follow‐up may limit the understanding of (dis)continuation of PrEP in those women not returning for a follow‐up visit. Lastly, the inability to blind women participating in the RCTs, due to the nature of the study interventions, may have overestimated adherence due to social desirability bias, particularly in studies using self‐reported PrEP use only as outcome measures.

## 5 CONCLUSIONS

This systematic review provides essential insights into PrEP use among pregnant and postpartum women, showing promising initiation within ANC/PNC services, but substantial challenges in continuation and adherence, with self‐reported measures overestimating true adherence. The variability in adherence highlights a need for more person‐centred, supportive interventions to ensure effective HIV prevention during pregnancy and the postpartum period. Long‐acting PrEP options may better align with women's preferences and overcome adherence challenges associated with daily oral regimens. To ensure equitable access to these innovations, global funding mechanisms must continue to prioritize maternal PrEP as a core component of HIV prevention efforts. Expanding access to long‐acting PrEP options through differentiated models of delivery, including pharmacies and community delivery, could help improve continuity and reduce the risk of HIV acquisition. To eliminate vertical transmission and end the HIV epidemic, it will be critical to safeguard against reductions in global health funding by ensuring sustained international investment and strengthened national commitment to maternal HIV prevention.

## COMPETING INTERESTS

DJD received funding from Gilead and ViiV Healthcare. SB received funding from MSD. All other authors have declared no conflicts of interest.

## AUTHORS' CONTRIBUTIONS

AR and BH conceived the study. AR and ZE did the literature search and bias assessment. DJD/BH reviewed for conflict. AR did data extraction and drafted the primary draft of the manuscript. AR and ZE did the risk of bias assessment. Discrepancies were resolved by DJD/BH. AR, ZE, SB, DJD and BH contributed critically to the manuscript and revisions, and approved the final version of the manuscript.

## FUNDING

DJD received funding from NICHD for this study (R01HD106862).

## Supporting information




**Appendix 1**: search strategies per database

## Data Availability

All data supporting the findings of this review were extracted from publicly available sources. The specific sources of the data have been cited within the manuscript. Due to the nature of the systematic review, no new data were created or collected. Detailed search strategies are available in the Supplementary Appendix.
