# Supplementary material for: Oral pre‐exposure prophylaxis initiation, continuation and adherence among pregnant and postpartum women receiving antenatal and postnatal care: a systematic review
Source: J Int AIDS Soc. 2025 Oct 8;28(Suppl 5):e70035. doi: 10.1002/jia2.70035 (PMC12505019; doi:10.1002/jia2.70035)
Supplement: Supplementary file 1 — Appendix 1: search strategies per database [file JIA2-28-e70035-s001.docx]

**Appendix to**

**Oral pre-exposure prophylaxis initiation, continuation, and adherence among pregnant and postpartum women attending antenatal and postnatal care: a systematic review**

*Authors*

Anke Rotsaert^1§^, Zaynab Essack^2,3^, Shannon Bosman^2^, Dvora Joseph Davey^4,5^, Bernadette Hensen^1^

*Affiliation*

1. Department of Public Health, Institute of Tropical Medicine, Antwerp, Belgium
2. Centre for Community-Based Research, Human Sciences Research Council, Pietermaritzburg, South Africa
3. South African Research Ethics Training Initiative, University of KwaZulu-Natal Natal, South Africa
4. Department of Infectious Diseases, Geffen School of Medicine, University of California, Los Angeles, United States
5. Department of Epidemiology & Biostatistics, School of Public Health, University of Cape Town, South Africa

§ *Corresponding Author*: Anke Rotsaert

Department of Public Health

Institute of Tropical Medicine

Nationalestraat 155

Antwerp, 2000, Belgium

+32497415860

arotsaert@itg.be

**Appendix 1: search strategies per database**

**Pubmed**

(hiv[Title/Abstract] OR "human immunodeficiency virus"[Title/Abstract] OR HIV[MeSH Terms]) AND ("postpartum period"[Title/Abstract] OR postpartum [Title/Abstract] OR pregnan* [Title/Abstract] OR perinatal [Title/Abstract] OR breastfeed*[Title/Abstract] OR lactati* [Title/Abstract] OR postpartum period [MeSH Terms] OR pregnancy [MeSH Terms]) AND (wom?n [Title/Abstract] OR "adolescent girl" [Title/Abstract] OR "adolescent girls" [Title/Abstract] OR "young women" [Title/Abstract] OR "young woman" [Title/Abstract] OR female [Title/Abstract] OR female [MeSH Terms]) AND ("pre-exposure prophylaxis" [Title/Abstract] OR "preexposure prophylaxis" [Title/Abstract] OR "prep" [Title/Abstract] OR Pre-Exposure Prophylaxis* [MeSH Terms])

+ filter: humans, English, start year 2015 🡪 306 results

**Web of Science Core Collection**

(TS=hiv OR TS="human immunodeficiency virus") AND (TS="postpartum period" OR TS=postpartum OR TS=pregnan* OR TS=perinatal OR TS=breastfeed* OR TS=lactati*) AND (TS=wom?n OR TS="adolescent girl" OR TS="adolescent girls" OR TS="young women" OR TS="young woman" OR TS=female) AND (TS="pre-exposure prophylaxis" OR TS="preexposure prophylaxis" OR TS= "prep")

+ filter: English, start year 2015 🡪 398 results

**Global Health**

[[[Publication Title: hiv] OR [Publication Title: "human immunodeficiency virus"]] AND [[Publication Title: "postpartum period"] OR [Publication Title: postpartum] OR [Publication Title: pregnan*] OR [Publication Title: perinatal] OR [Publication Title: breastfeed*] OR [Publication Title: lactati*]] AND [[Publication Title: wom?n] OR [Publication Title: "adolescent girl"] OR [Publication Title: "adolescent girls"] OR [Publication Title: "young women"] OR [Publication Title: "young woman"] OR [Publication Title: female]] AND [[Publication Title: "pre-exposure prophylaxis"] OR [Publication Title: "preexposure prophylaxis"] OR [Publication Title: "prep"]]] OR [[[ab: hiv] OR [ab: "human immunodeficiency virus"]] AND [[ab: "postpartum period"] OR [ab: postpartum] OR [ab: pregnan*] OR [ab: perinatal] OR [ab: breastfeed*] OR [ab: lactati*]] AND [[ab: wom?n] OR [ab: "adolescent girl"] OR [ab: "adolescent girls"] OR [ab: "young women"] OR [ab: "young woman"] OR [ab: female]] AND [[ab: "pre-exposure prophylaxis"] OR [ab: "preexposure prophylaxis"] OR [ab: "prep"]]] AND [Publication Date: (01/01/2015 TO 03/31/2024)]

+ filter English 🡪 164 results
